# Reliability, Validity, and Factor Structure of the Internalized AIDS-Related Stigma Scale in Southern India

**DOI:** 10.1177/2325958219831025

**Published:** 2019-02-20

**Authors:** Brian T. Chan, Amrose Pradeep, Ezhilarasi Chandrasekaran, Lakshmi Prasad, Vinothini Murugesan, Nagalingeswaran Kumarasamy, Kenneth H. Mayer, Alexander C. Tsai

**Affiliations:** 1 Division of Infectious Diseases, Brigham and Women’s Hospital, Boston, MA, USA; 2 Harvard Medical School, Boston, MA, USA; 3 Y. R. Gaitonde Centre for AIDS Research and Education, Chennai, India; 4 Division of Infectious Diseases, Beth Israel Deaconess Medical Center, Boston, MA, USA; 5 Fenway Health, Boston, MA, USA; 6 MGH Global Health, Massachusetts General Hospital, Boston, MA, USA; 7 Chester M. Pierce, MD Division of Global Psychiatry, Massachusetts General Hospital, Boston, MA, USA

**Keywords:** internalized stigma, India, validation

## Abstract

We used data from 660 people living with HIV in southern India to evaluate the reliability, validity, and factor structure of the Internalized AIDS-Related Stigma Scale. Exploratory factor analysis revealed the presence of 2 factors: a 2-item factor related to disclosure concerns and a 4-item factor related to self-hatred. The self-hatred factor demonstrated acceptable internal consistency (Cronbach α = .80). As evidence of construct validity, both factors were correlated with depression symptom severity as measured by the Patient Health Questionnaire-9. Further study is needed to understand the correlates of these factors and their impact on the Indian HIV care continuum.

**What Do We Already Know about This Topic?**

HIV-related stigma, the social discrediting or devaluation associated with HIV, remains a major impediment to prevention and treatment initiatives worldwide and specifically in India, which has the world’s third largest HIV epidemic.

**How Does Your Research Contribute to the Field?**

In this assessment of the factor structure, reliability (internal consistency), and construct validity of the 6-item Internalized AIDS-Related Stigma Scale (IARSS) in a sample of people living with HIV in southern India, we found evidence for 2 distinct factors related to disclosure concerns and self-hatred.

**What Are Your Research’s Implications toward Theory, Practice, or Policy?**

Although the IARSS may be useful in the Indian context, more study is needed to understand the correlates of the disclosure concerns and self-hatred factors and the impact these factors may have on the Indian HIV care continuum.

## Introduction

HIV-related stigma, the social discrediting or devaluation associated with HIV, remains a major impediment to prevention and treatment initiatives worldwide. Internalized stigma occurs when members of a stigmatized group internalize and accept negative attitudes held by the majority group, leading to the development of self-defacing beliefs.^1^ Among people living with HIV (PLHIV), internalized stigma has been associated with worsened outcomes such as poor mental health^2 ,3^ and suboptimal antiretroviral therapy (ART) adherence.^4^ In India, where ART scale-up continues to be hampered by high levels of HIV-related stigma,^5^ there is need for a validated internalized stigma scale brief enough to be used in common clinical and research settings. HIV-related stigma scales validated previously in India have focused on stigma in the community^6^ or targeted multiple dimensions of stigma.^7^ As such, we used data from southern India to assess the factor structure, reliability (internal consistency), and construct validity of an internalized stigma scale previously used in settings such as southern^8^ and eastern Africa.^9^

## Methods

### Study Setting and Procedures

Located in Chennai, Tamil Nadu state, the Y. R. Gaitonde Centre for AIDS Research and Education (YRG CARE) is one of the largest private organizations providing HIV care in India, having treated more than 20 000 PLHIV since its founding in 1993. Patients from all over India seek care at YRG CARE, although a majority of patients are from Tamil Nadu and Andhra Pradesh states. Historically, most patients at YRG CARE have self-identified as heterosexual,^10^ as there are other local organizations in Chennai perceived as specializing in care for sexual and gender minorities.

As part of a parent study on prevalence and correlates of psychosocial conditions among Indian PLHIV,^10^ we collected data on internalized stigma and depression on a convenience sample of patients 18 years of age and older who were presenting for their first clinical visit after HIV diagnosis between January 2015 and November 2016. Study materials were written in English, professionally translated into Tamil or Telugu and back-translated by different professional translators (a total of 4 translators). We scrutinized the back-translations and found no important discrepancies between the original English and back-translated versions. We pilot-tested the questionnaire on 5 Tamil and 5 Telugu speakers to ensure comprehensibility, face and content validity, and conceptual equivalence between the English and Tamil/Telugu versions. Interviews were conducted face-to-face in the patient’s preferred language by 1 of 2 female HIV counselors.

### Ethical Consideration and Informed Consent

We obtained signed or thumbprint-marked (in cases of illiteracy) informed consent documents from all participants. Ethical approval was obtained from the Institutional Review Boards of Fenway Health (approval no. 483376) and YRG CARE (approval no. YRG-265).

### Measures

We focused our analysis on the 6-item Internalized AIDS-Related Stigma Scale (IARSS),^8^ which includes items related to concerns about disclosure as well as items related to feelings of shame and/or self-hatred. Responses are elicited on a dichotomic scale (agree/disagree) and scores represent the sum of endorsed items. We assessed for depressive symptoms using the Patient Health Questionnaire-9 (PHQ-9), which has been previously validated in Indian settings and translated into Tamil and Telugu.^11^ Sociodemographic variables of interest included gender, age, educational attainment, marital status, employment status, rural/urban residence, language (Tamil/Telugu/other), sexual orientation, alcohol use (“yes” versus “no” or “stopped”), and injection drug use.

### Statistical Analysis

As the IARSS was developed to measure one dimension of stigma (internalized stigma),^8^ we sought to assess its dimensionality by performing exploratory factor analysis (EFA) on the IARSS items using principal-factors extraction and orthogonal varimax rotation, returning all factors with eigenvalues >1.0. We then graphed the eigenvalues in decreasing order to identify the scree, if any. Next, we examined the loadings of the individual items on the factor(s), assigning an item to a factor if its factor loading was ≥0.40. To assess the internal consistency of any identified factors, we calculated Cronbach α, using 2000 bootstrap replications to compute the standard errors. We examined item–test correlations and recalculated the Cronbach α after sequentially deleting individual items. We then conducted the EFA stratified by gender.

We did not have access to a gold standard to assess criterion-related validity. Instead, we used the PHQ-9 score to assess construct validity of the factor(s), as an association between internalized stigma and poor mental health status has been demonstrated in multiple settings.^2 ,3^ We fitted linear regression models, adjusted for sociodemographic variables, specifying the factor(s) as the exposure and PHQ-9 score (0-27) as the outcome. A statistically significant regression coefficient was considered evidence that an association existed between the factor(s) and depressive symptoms. In addition, for any factors with multiple items, we calculated the Spearman correlation coefficient between the factor(s) and the PHQ-9 score. All analyses were performed using Stata software (version 13.1, StataCorp, College Station, Texas).

## Results

We collected data on 660 persons, including 388 men, 268 women, and 4 Hijras (transgender women; Table 1). None of the 660 persons who were approached to enter the study refused entry into the study and all participants completed the questionnaire. A plurality of participants (296, 44.9%) responded affirmatively to 2 stigma items. A total of 119 (18.0%) responded negatively to all 6 items while 34 (5.2%) responded affirmatively to all 6 items. Exploratory factor analysis revealed 2 factors with eigenvalues of 2.45 and 1.97. There was no clear scree noted on the scree plot. The 4 items related to self-hatred or shame loaded positively on the first factor, while the 2 items related to fears of disclosure loaded positively on the second factor (Table 2). The Cronbach α of the self-hatred factor was .80 (95% confidence interval [CI]: 0.77-0.83). Item-test correlations were approximately equal for each of the 4 items. In analyses stratified by gender, results were similar between men and women (results available in Supplementary Digital Content).

**Table 1.** Sample Characteristics (n = 660).

	Median (Interquartile Range) or No. (%)
Age, years	39 (34-45)
Gender	
Male	388 (59%)
Female	268 (41%)
Hijra (transgender women)	4 (0.6%)
Achieved more than primary education	441 (67%)
Married or cohabiting	451 (68%)
Currently employed	496 (75%)
Urban residence	221 (34%)
Tamil speaker	123 (19%)
Telugu speaker	507 (77%)
Heterosexual	631 (96%)
Current alcohol use	97 (15%)
Injection drug use	3 (0.5%)
PHQ-9 score	2 (0-4)
Internalized AIDS-Related Stigma Scale (IARSS) score	2 (2-3)

Abbreviation: PHQ-9, Patient Health Questionnaire-9.

**Table 2.** Internalized AIDS-Related Stigma Scale with 2 Factors.

Item	No. (%) or Mean (SD)	Factor Loading	Item–Test Correlation	Cronbach α if Deleted
Factor 1: Self-hatred				
Being HIV positive makes me feel dirty	175 (26.5%)	0.62	0.76	.79
I feel guilty that I am HIV positive	156 (23.6%)	0.82	0.86	.68
I am ashamed that I am HIV positive	71 (10.8%)	0.84	0.76	.74
I sometimes feel worthless because I am HIV positive	92 (13.9%)	0.82	0.78	.73
*Endorsed at least one self-hatred item*	225 (35.8%)			
*Mean self-hatred score (0*-*4)*	0.8 (1.2)			
Factor 2: Fears of disclosure				
It is difficult to tell people about my HIV infection	533 (80.8%)	0.95	n/a	n/a
I hide my HIV status from others	517 (78.3%)	0.95	n/a	n/a
*Endorsed at least one fears of disclosure item*	540 (81.8%)			
*Mean fears of disclosure score (0*-*2)*	1.6 (0.8)			

Abbreviation: SD, standard deviation.

The Spearman correlation coefficient between self-hatred and PHQ-9 score was 0.41 (95% CI: 0.37-0.45), indicating a correlation of moderate magnitude. This correlation was consistent with the findings of a linear regression model fitted to the data. We estimated a statistically significant positive association between self-hatred (a variable equal to the number of affirmative responses to the 4 self-hatred items) and PHQ-9 score (adjusted *b =* 1.13; 95% CI: 0.94-1.32). Turning next to the second factor, the Spearman correlation coefficient between fears of disclosure and PHQ-9 score was 0.26 (95% CI: 0.23-0.29). In a linear regression model with fears of disclosure as the exposure, we estimated a statistically significant positive association between fears of disclosure and PHQ-9 score (adjusted *b =* 1.75; 95% CI: 1.13-2.37).

## Discussion

In this analysis of data from PLHIV in Chennai, India, we assessed the factor structure, reliability, and construct validity of the 6-item IARSS. We found evidence for a 2-item factor related to disclosure concerns and a 4-item factor related to self-hatred. Furthermore, we found that PLHIV in our sample were much more likely to endorse fears of disclosure than feelings of self-hatred. These findings contrast IARSS validation studies conducted in other settings, such as Uganda, which supported a unidimensional factor structure.^8 ,9^

Compared to the generalized epidemics found in sub-Saharan Africa, the Indian HIV epidemic is concentrated among key groups. As such, it is possible that intensity of stigmatizing attitudes among the general population in India is relatively low, leading to relatively little internalization of self-hatred among PLHIV. However, while Indian PLHIV may not accept stigmatizing attitudes toward PLHIV as valid, in an environment characterized by a high degree of structural violence and social/legal discrimination directed toward sexual and gender minorities,^12-14^ they may fear consequences of disclosure such as discrimination or violence. This may be consistent with studies which have shown that Indian PLHIV anticipate stigma much more frequently than they actually experience enactments of stigma.^5^ In this regard, the items related to disclosure concerns may be more closely linked to the construct of anticipated stigma, defined as the expectation that persons with a stigmatized attribute will be devalued and subjected to discrimination.^15^

We found that both the disclosure concerns and self-hatred factors were associated with depression symptom severity, evidence for the construct validity of these factors. The scale items for the 4-item self-hatred factor had a high internal consistency and the magnitude of the correlation between the self-hatred factor and PHQ-9 score was similar to the magnitude of the correlation between IARSS and depression found in the original IARSS validation study.^8^ The correlation between disclosure concerns and PHQ-9 was relatively weak, which we believe makes sense given that the drivers of disclosure concerns are likely to be rooted more in societal factors (such as the presence of stigmatizing attitudes in the general population) rather than an individual’s level of depression symptoms. In total, our findings suggest that while the IARSS may be useful, it captures concepts of fears of disclosure and self-hatred that are likely to be distinct phenomena in India. Confirmatory factor analysis to test and confirm this factorial structure would be a worthwhile extension for future study.

Our study has several limitations. First, our sample was a convenience sample of PLHIV presenting for HIV care. As stigma has been associated with lower rates of HIV testing^16^ and care-seeking,^17^ the participants in our sample are likely to have had lower levels of internalized stigma compared to PLHIV who are out of care. Second, this study was conducted at a single center where most patients self-identify as heterosexual; thus, the findings may not be generalizable to the entire country. However, the type of private, multiservice program exemplified by YRG CARE can be found in many other Indian cities, as well as other countries in Asia. Future studies should be undertaken at ART centers with greater numbers of sexual and gender minorities, especially given the intersectionality of HIV-related stigma with gender identity and sexual orientation stigmas. Third, we could not establish criterion-related validity, as we did not have access to a gold standard measure of internalized stigma. However, this limitation appears to be inherent to validation studies of internalized stigma scales. Finally, in the interest of limiting the length of the survey instrument, this study focused on internalized stigma and did not address other stigma dimensions. However, there exists a growing consensus that internalized stigma is a particularly important predictor of poor HIV-related outcomes.^4^

## Conclusion

We assessed the factor structure, reliability (internal consistency), and construct validity of the 6-item IARSS in a sample of PLHIV in southern India. We found evidence for 2 distinct factors related to disclosure concerns and self-hatred. Although the IARSS may be useful in the Indian context, more study is needed to understand the correlates of these factors and the impact these factors may have on the Indian HIV care continuum.

## Supplemental Material

Supplemental Material, IARSSIndia-JIAPAC_revised_Supplemental_material - Reliability, Validity, and Factor Structure of the Internalized AIDS-Related Stigma Scale in Southern India Click here for additional data file. Supplemental Material, IARSSIndia-JIAPAC_revised_Supplemental_material for Reliability, Validity, and Factor Structure of the Internalized AIDS-Related Stigma Scale in Southern India by Brian T. Chan, Amrose Pradeep, Ezhilarasi Chandrasekaran, Lakshmi Prasad, Vinothini Murugesan, Nagalingeswaran Kumarasamy, Kenneth H. Mayer, and Alexander C. Tsai in Journal of the International Association of Providers of AIDS Care (JIAPAC)

## References

[1] Link BG Cullen FT Struening E Shrout PE Dohrenwend BP A modified labeling theory approach to mental disorders: an empirical assessment. Am Sociol Rev. 1989;54(3):400 doi:10.2307/2095613.

[2] Simbayi LC Kalichman S Strebel A Cloete A Henda N Mqeketo A Internalized stigma, discrimination, and depression among men and women living with HIV/AIDS in Cape Town, South Africa. Soc Sci Med. 2007;64(9):1823–1831. doi:10.1016/j.socscimed.2007.01.006. 17337318 10.1016/j.socscimed.2007.01.006 PMC4271649

[3] Tsai AC Bangsberg DR Frongillo EA Food insecurity, depression and the modifying role of social support among people living with HIV/AIDS in rural Uganda. Soc Sci Med. 2012;74(12):2012–2019. doi:10.1016/j.socscimed.2012.02.033. 22513248 10.1016/j.socscimed.2012.02.033 PMC3348339

[4] Katz IT Ryu AE Onuegbu AG Impact of HIV-related stigma on treatment adherence: systematic review and meta-synthesis. J Int AIDS Soc. 2013;16(3 suppl 2):18640. 24242258 10.7448/IAS.16.3.18640 PMC3833107

[5] Steward WT Herek GM Ramakrishna J HIV-related stigma: adapting a theoretical framework for use in India. Soc Sci Med. 2008;67(8):1225–1235. doi:10.1016/j.socscimed.2008.05.032. 18599171 10.1016/j.socscimed.2008.05.032 PMC2603621

[6] Zelaya CE Sivaram S Johnson SC Srikrishnan AK Solomon S Celentano DD HIV/AIDS stigma: reliability and validity of a new measurement instrument in Chennai, India. AIDS Behav. 2008;12(5):781–788. doi:10.1007/s10461-007-9331-7. 18030613 10.1007/s10461-007-9331-7

[7] Jeyaseelan L Kumar S Mohanraj R Rebekah G Rao D Manhart LE Assessing HIV/AIDS stigma in south India: validation and abridgement of the Berger HIV Stigma scale. AIDS Behav. 2013;17(1):434–443. doi:10.1007/s10461-011-0128-3. 22246514 10.1007/s10461-011-0128-3 PMC3404245

[8] Kalichman SC Simbayi LC Cloete A Mthembu PP Mkhonta RN Ginindza T Measuring AIDS stigmas in people living with HIV/AIDS: the Internalized AIDS-Related Stigma Scale. AIDS Care. 2009;21(1):87–93. doi:10.1080/09540120802032627. 19085224 10.1080/09540120802032627

[9] Tsai AC Weiser SD Steward WT Evidence for the reliability and validity of the Internalized AIDS-Related Stigma Scale in rural Uganda. AIDS Behav. 2013;17(1):427–433. doi:10.1007/s10461-012-0281-3. 22869104 10.1007/s10461-012-0281-3 PMC3505811

[10] Chan BT Pradeep A Prasad L Prevalence and correlates of psychosocial conditions among people living with HIV in southern India. AIDS Care. 2016;29(6):746–750. doi:10.1080/09540121.2016.1231887. 27643850 10.1080/09540121.2016.1231887 PMC5552362

[11] Kroenke K Spitzer RL The PHQ-9: a new depression diagnostic and severity measure. Psychiatr Ann. 2002;32(9):509–515. doi:10.3928/0048-5713-20020901-06.

[12] Bharat S A systematic review of HIV/AIDS-related stigma and discrimination in India: current understanding and future needs. SAHARA J. 2011;8(3):138–149. doi:10.1080/17290376.2011.9724996. 23237728 10.1080/17290376.2011.9724996

[13] Chakrapani V Newman PA Shunmugam M McLuckie A Melwin F Structural violence against Kothi-identified men who have sex with men in Chennai, India: a qualitative investigation. AIDS Educ Prev. 2007;19(4):346–364. doi:10.1521/aeap.2007.19.4.346. 17685847 10.1521/aeap.2007.19.4.346

[14] Thomas B Kumar S Safren S Mimiaga M Swaminathan S Mayer K HIV in Indian MSM: reasons for a concentrated epidemic & strategies for prevention. Indian J Med Res. 2011;134(6):920 doi:10.4103/0971-5916.92637. 22310824 10.4103/0971-5916.92637 PMC3284100

[15] Link BG Understanding labeling effects in the area of mental disorders: an assessment of the effects of expectations of rejection. Am Sociol Rev. 1987;52(1):96–112.

[16] Kalichman SC Simbayi LC HIV testing attitudes, AIDS stigma, and voluntary HIV counselling and testing in a black township in Cape Town, South Africa. Sex Transm Infect. 2003;79(6):442–447. 14663117 10.1136/sti.79.6.442 PMC1744787

[17] Murray LK Semrau K McCurley E Barriers to acceptance and adherence of antiretroviral therapy in urban Zambian women: a qualitative study. AIDS Care. 2009;21(1):78–86. doi:10.1080/09540120802032643. 19085223 10.1080/09540120802032643 PMC2821879

