# Supplementary material for: Reliability, Validity, and Factor Structure of the Internalized AIDS-Related Stigma Scale in Southern India
Source: J Int Assoc Provid AIDS Care. 2019 Feb 20;18:2325958219831025. doi: 10.1177/2325958219831025 (PMC6748476; doi:10.1177/2325958219831025)
Supplement: Supplemental Material, IARSSIndia-JIAPAC_revised_Supplemental_material - Reliability, Validity, and Factor Structure of the Internalized AIDS-Related Stigma Scale in Southern India [file IARSSIndia-JIAPAC_revised_Supplemental_material.pdf]

Supplementary Digital Content

Internalized AIDS-Related Stigma Scale with two factors, men only

Item	No. (%) or mean (SD)	Factor loading	Item-test correlation	Cronbach's alpha if deleted
Factor 1: Self-hatred				
Being HIV positive makes me feel dirty	109 (28.1%)	0.65	0.77	0.80
I feel guilty that I am HIV positive	95 (24.5%)	0.83	0.85	0.70
I am ashamed that I am HIV positive	47 (12.1%)	0.84	0.78	0.75
I sometimes feel worthless because I am HIV positive	62 (16.0%)	0.81	0.79	0.75
Endorsed at least one self-hatred item	85 (31.7%)			
Mean self-hatred score (0-4)	0.8 (1.3)			
Factor 2: Fears of disclosure				
It is difficult to tell people about my HIV infection	315 (81.2%)	0.95	n/a	n/a
I hide my HIV status from others	310 (79.9%)	0.96	n/a	n/a
Endorsed at least one fears of disclosure item	321 (82.7%)			
Mean fears of disclosure score (0-2)	1.6 (0.8)			

Internalized AIDS-Related Stigma Scale with two factors, women only

Item	No. (%) or mean (SD)	Factor loading	Item-test correlation	Cronbach's alpha if deleted
Factor 1: Self-hatred				
Being HIV positive makes me feel dirty	65 (24.3%)	0.56	0.75	0.79
I feel guilty that I am HIV positive	60 (22.4%)	0.80	0.87	0.65
I am ashamed that I am HIV positive	24 (9.0%)	0.83	0.74	0.73
I sometimes feel worthless because I am HIV positive	30 (11.2%)	0.85	0.77	0.71
Endorsed at least one self-hatred item	225 (34.1))			
Mean self-hatred score (0-4)	0.7 (1.1)			

Factor 2: Fears of disclosure				
It is difficult to tell people about my HIV infection	215 (80.2%)	0.95	n/a	n/a
I hide my HIV status from others	204 (76.1%)	0.95	n/a	n/a
Endorsed at least one fears of disclosure item	216 (80.6%)			
Mean fears of disclosure score (0-2)	1.6 (0.8)			
